# Alteration of inflammatory cytokine production in primary total knee arthroplasty using antibiotic-loaded bone cement

**DOI:** 10.1007/s10195-016-0432-9

**Published:** 2016-10-21

**Authors:** Vajara Wilairatana, Peerasit Sinlapavilawan, Sittisak Honsawek, Noppachart Limpaphayom

**Affiliations:** 1 Department of Orthopaedics, Faculty of Medicine, Chulalongkorn University, Bangkok, 10330 Thailand; 2 Department of Biochemistry, Faculty of Medicine, Chulalongkorn University, Bangkok, 10330 Thailand

**Keywords:** Assessment, Patient outcome, Bone cements, C-reactive protein, Erythrocyte sedimentation rate, Inflammation, Interleukin-6, Knee arthroplasty

## Abstract

**Background:**

The aim was to compare serum levels of interleukin-6 (SIL6), erythrocyte sedimentation rate (ESR), C-reactive protein (CRP) and joint fluid IL-6 (JIL-6) level between total knee arthroplasty (TKA) that used bone cement (BC group) and antibiotic-loaded bone cement (ALBC group).

**Materials and methods:**

Thirty-nine patients (40 TKAs) with a mean age of 69.6 years were non-randomly assigned to the BC or ALBC groups. Samples of SIL6, ESR, and CRP were collected at baseline and at 24 h, 72 h, and 4 weeks after surgery. JIL-6 levels were collected intraoperatively before joint arthrotomy, before finishing the procedure and 24 h after surgery. Postoperative mediator levels were compared with baseline levels within the same group and between groups at each time point. Knee Society Score-knee (KSSK) and Knee Society Score-function (KSSF) scores were evaluated.

**Results:**

SIL-6, ESR, and CRP levels were significantly elevated at 24 and 72 h, compared to baseline. Only SIL-6 levels at 72 h in the ALBC group showed a significantly lower level than those in the BC group. JIL-6 levels were not different between groups. There were no significant differences in KSSK or KSSF scores between groups at 4 weeks and the most recent follow-up (1.4 ± 0.6 years) evaluation. No correlations were identified among SIL-6 and JIL-6 levels at 24 and 72 h, and KSSK and KSSF scores at 4 weeks and at the most recent evaluation.

**Conclusions:**

ALBC showed a favorable immunomodulatory effect and lower SIL-6 level at 72 h following TKA compared to BC, although functional benefits require further investigation.

**Level of evidence:**

OCEBM, Level 3.

## Introduction

Total knee arthroplasty (TKA) using bone cement is a standard treatment for severe osteoarthritis (OA) of the knee joint. Immunologic changes demonstrated by increasing levels of proinflammatory cytokines [e.g., interleukin-6 (IL-6)] after the procedure are well-recognized and could be activated by various stimuli, including nociceptive stimulation, tissue injury, ischemia or reperfusion, and hemodynamic disturbances [1–3]. Several modalities for decreasing postoperative inflammation to improve an outcome have been studied [4, 5].

IL-6 is a well-established inflammatory marker associated with TKA procedure [3]. High joint fluid IL-6 (JIL-6) levels correlated with functional outcome of TKA [6] and were linked to postoperative arthrofibrosis [7]. Moreover, serum IL-6 (SIL-6) was found to control C-reactive protein (CRP) release and alter erythrocyte sedimentation rate (ESR) and, therefore, could be a predictor of TKA outcome [8]. Accordingly, controlling the IL-6 level after TKA may have a beneficial effect in controlling postoperative inflammation.

Antibiotics impregnated in bone cement powder deliver a high level of antibiotics locally and a detectable level of antibiotics systemically [9–11]. Recent studies have supported the routine use of antibiotic-loaded bone cement (ALBC) for prophylactic purposes in cases of primary TKA [12, 13]. Moreover, the non-antibacterial properties of antibiotics are recognized as exhibiting immunomodulatory effects and helping to regulate body inflammatory response [14]. In vitro studies showed that gentamicin could alter SIL-6 levels [14, 15]; however, the in vivo effect of commercially available gentamicin-loaded bone cement on systemic and local proinflammatory cytokines and acute-phase proteins in TKA has not been studied.

We hypothesized that ALBC could alter proinflammatory cytokine levels and modify clinical outcomes in patients who underwent primary TKA. As such, the objectives of this study were (1) to compare serum levels of IL-6, ESR, and CRP and JIL-6 level in patients who underwent primary TKA using two different types of bone cement, and (2) to evaluate the association between IL-6 levels and functional outcomes.

## Materials and methods

Thirty-nine patients with primary knee OA who required TKA were enrolled. The study subjects included 3 males and 36 females with an average age of 69.6 ± 7.2 years (range 54–84). Patients with a history of inflammatory or infective joint disease, systemic infection, and patients with suspected immunocompromised status were excluded. All TKA procedures were performed by a single surgeon (VW). Standard medial parapatellar approach TKA with patellar resurfacing under tourniquet control was performed using the posterior-stabilized knee system as follows—Scorpio NRG^®^ (Stryker Corporation, Kalamazoo, MI, USA) in 3 knees, Nexgen LPS-Flex^®^ (Zimmer, Warsaw, IN, USA) in 9 knees, and VEGA^®^ (B. Braun Melsungen AG, Melsungen, Germany) in 28 knees.

The type of bone cement was prepared in a sealed opaque envelope, which was opened by a surgical nurse immediately prior to implantation of the device in a non-randomized fashion. Bone cement was hand-mixed in an open bowl in every case. Palacos^®^ R (Heraeus Medical GmbH, Wehrheim, Germany) was used in the bone cement without antibiotics (BC group) and Palacos^®^ R + G (Heraeus Medical GmbH, Wehrheim, Germany) was used in the ALBC group.

The postoperative protocol was consistent among all patients. The closed-suction tube was removed at 24 h after surgery. The operated knee was immobilized for 72 h, after which range of motion and progressive weight-bearing exercises were allowed. Knee Society Score-knee (KSSK) and Knee Society Score-function (KSSF) scores were evaluated at 4 weeks after surgery and at the most recent follow-up visit [16].

### Determination of IL-6, ESR, and CRP levels

SIL-6, ESR, and CRP levels were measured upon admission (within 24 h before TKA procedure) and at 24 h, 72 h, and 4 weeks after surgery. JIL-6 levels were obtained during the TKA procedure by aspiration of fluid from the knee joint before arthrotomy, at joint space closure and at 24 h after surgery via clamped suction tube drainage.

All samples were immediately placed on ice for transfer. Samples were centrifuged at 1000*g* for 15 min to remove cells and serum samples were stored at −80 °C until analysis. IL-6 concentration was determined by human enzyme-linked immunosorbent assay (LEGEND MAX™ Human IL-6 ELISA Kit; BioLegend, Inc., San Diego, CA, USA) using standard concentration-optical density curve. ESR level was analyzed using a Monitor-20 ESR analyzer (Vital Diagnostics, Lincoln, RI, USA) and CRP level was determined using BN ProSpec^®^ System (Siemens Healthcare GmbH, Erlangen, Germany).

### Statistical analysis

Studied parameters were checked for normal distribution by Shapiro–Wilk test. Demographic data were expressed as mean ± standard deviation (SD) and serum and joint fluid mediator levels as median (interquartile range, IQR). Student’s *t* test or Pearson’s chi-square test was used to analyze demographic data between groups when appropriate. Differences in SIL-6, ESR, CRP, and JIL-6 levels between baseline and each subsequent time point within the same bone cement group were compared using Wilcoxon signed-rank test. At each time point, mediator levels were compared between the two bone cement groups by Wilcoxon rank-sum test. KSSK and KSSF scores were evaluated between groups at 4 weeks and at the most recent follow-up by Wilcoxon rank-sum test. Spearman’s rank correlation coefficient (Spearman’s rho, ρ) was used to identify associations among SIL-6 and JIL-6 levels, KSSK, and KSSF at 4 weeks and at the most recent follow-up. A *p* value of <0.05 was considered statistically significant. Statistical analyses were performed using Stata 12.0 software (StataCorp LP, College Station, TX, USA).

This study was approved by the Institutional Review Board of the Faculty of Medicine, Chulalongkorn University (COA No.185/2013). This study complied with all provisions set forth in the Declaration of Helsinki (1964) and all of its subsequent amendments. Written informed consent was obtained from each subject prior to his/her participation in this study and the anonymity of all patients was protected and preserved.

## Results

Thirty-nine eligible patients (40 knees) were enrolled between June 2013 and December 2013. Preoperative Ahlbäck classification was class 3 in 4 knees and class 4 in 36 knees. Thirty-eight patients underwent unilateral TKA, with one patient having bilateral TKAs that were performed 6 months apart. Each bone cement group consisted of 20 knees. The distribution of types of prosthesis used was similar among BC and ALBC groups (chi-squared = 1.48, *p* = 0.48). Demographic and clinical data of both groups are shown in Table 1.

**Table 1:** Demographic and clinical data of the 40 total knee arthroplasties in this study

	BC group (*n* = 20)	ALBC group (*n* = 20)	*p*
Age (years)	71.2 ± 7.9 (54.0–84.0)	67.9 ± 6.2 (59.0–79.0)	0.15
Body weight (kg)	68.4 ± 11.4 (48.8–87.0)	70.1 ± 10.4 (53.6–90.0)	0.62
Height (m)	1.55 ± 0.1 (1.45–1.72)	1.55 ± 0.1 (1.39–1.69)	0.66
Body mass index (kg/m^2^)	28.1 ± 3.1 (21.7–33.3)	29.3 ± 3.9 (24.6–38.5)	0.27
Operative time (min)	99.5 ± 13.2 (90.0–120.0)	102.5 ± 14.1 (90.0–120.0)	0.49
Postoperative drainage (mL)	441.0 ± 128.1 (110.0–680.0)	389.0 ± 108.1 (160.0–600.0)	0.17

Data are presented as mean ± standard deviation (range)

*ALBC* antibiotic-loaded bone cement, *BC* bone cement

**Fig. 1:**
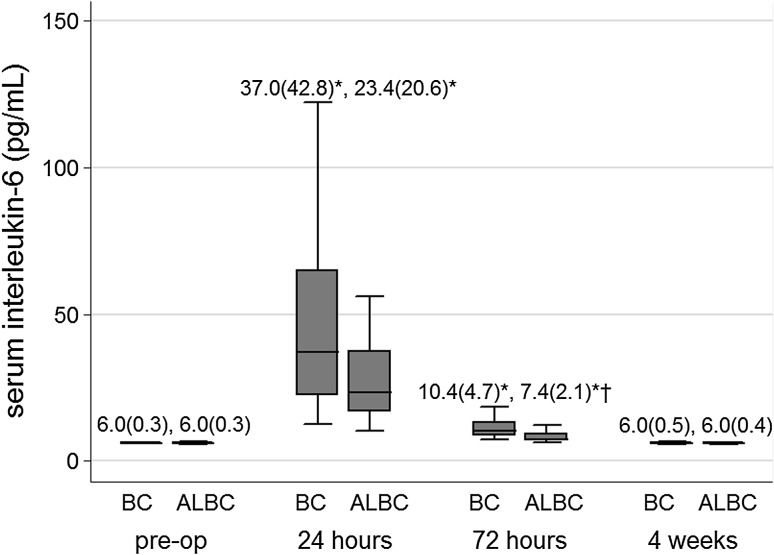
Serum interleukin-6 levels at each time point according to bone cement group. Data are presented as median (IQR). *p < 0.05 indicates statistically significant difference compared to preoperative baseline value within the same bone cement group; ^†^ *p* < 0.05 indicates statistically significant difference between the two bone cement groups at the same time point. *ALBC* antibiotic-loaded bone cement, *BC* bone cement

**Fig. 2:**
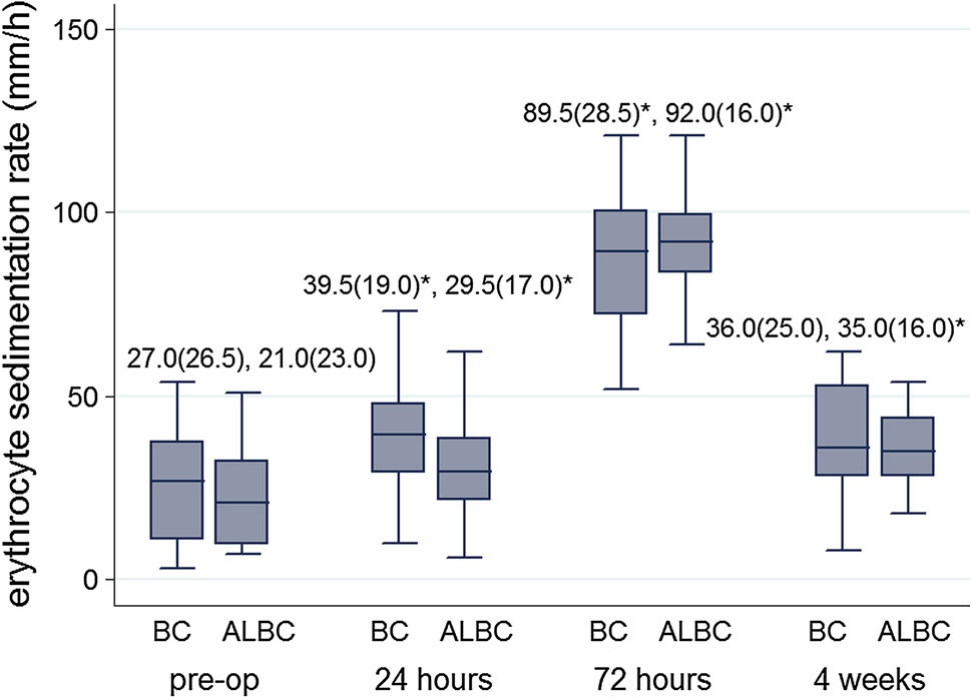
Erythrocyte sedimentation rate at each time point according to bone cement group. Data are presented as median (IQR). *p < 0.05 indicates statistically significant difference compared to preoperative baseline value within the same bone cement group. *ALBC* antibiotic-loaded bone cement, *BC* bone cement

**Fig. 3:**
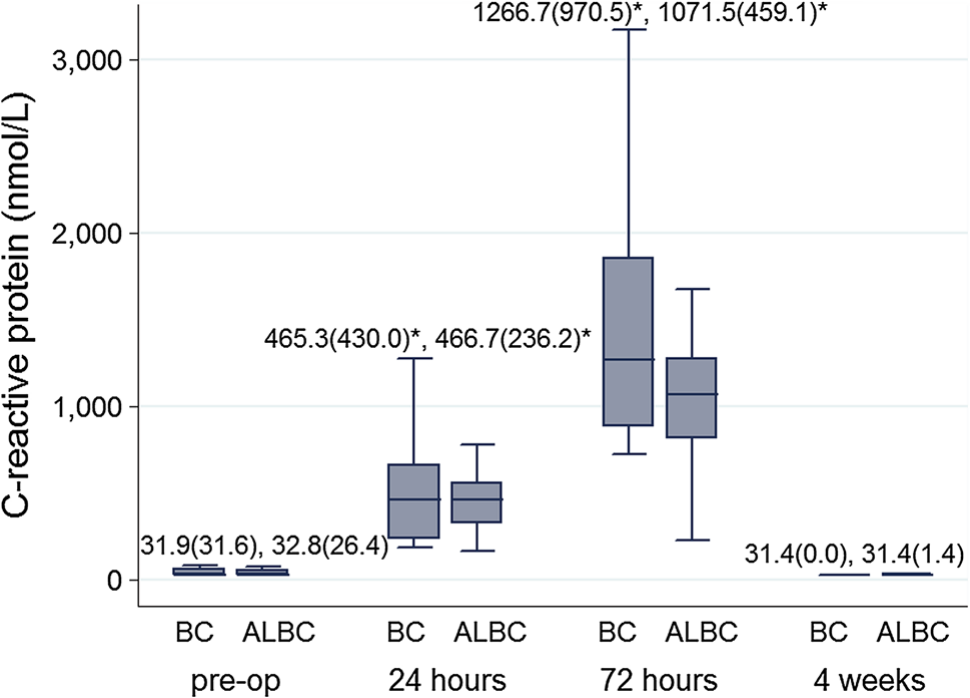
C-reactive protein at each time point according to bone cement group. Data are presented as median (IQR). *p < 0.05 indicates statistically significant difference compared to preoperative baseline value within the same bone cement group. *ALBC* antibiotic-loaded bone cement, *BC* bone cement

**Fig. 4:**
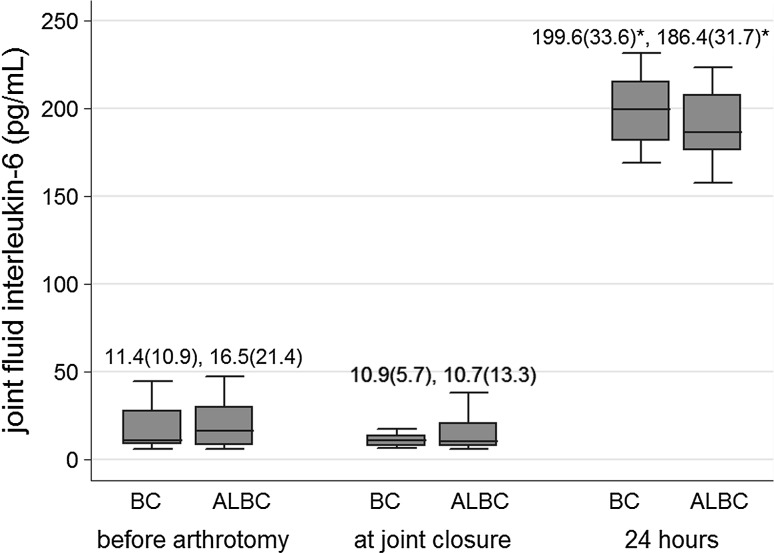
Joint fluid interleukin-6 levels at each time point according to bone cement group. Data are presented as median (IQR). *p < 0.05 indicates statistically significant difference compared to preoperative baseline value within the same bone cement group. *ALBC* antibiotic-loaded bone cement, *BC* bone cement

SIL-6 (Fig. 1), ESR (Fig. 2), and CRP (Fig. 3) levels increased significantly at 24 and 72 h from baseline levels in both bone cement groups. Median (IQR) SIL-6 levels peaked at 24 h, with the median level increase being higher in the BC group than in the ALBC group. The difference between groups, however, was not statistically significant [37.0 (42.8) vs 23.4 (20.6) pg/mL; *p* = 0.07]. At 72 h, SIL-6 levels in both groups showed a decreasing trend, but the median level descent was less in the BC group than in the ALBC group. Median (IQR) SIL-6 level remained at a significantly higher level in the BC group than in the ALBC group [10.4 (4.7) vs 7.4 (2.1) pg/mL; *p* = 0.001]. SIL-6 levels returned to their baseline preoperative levels at 4 weeks after surgery. ESR and CRP levels were not different between groups at 24 and 72 h after surgery. Serum levels of IL-6 and CRP in both groups, and ESR (in BC group) decreased to baseline levels at 4 weeks after surgery. Median (IQR) JIL-6 level was significantly elevated at 24 h compared to intraoperative level. However, although the JIL-6 level was higher in the BC group than in the ALBC group at this time point, there was no difference between the two cement groups [199.6 (33.6) vs 186.4 (31.7) pg/mL; *p* = 0.18] (Fig. 4).

There were no significant differences in KSSK and KSSF scores between bone cement groups at 4 weeks and at the most recent follow-up evaluation (average follow-up 1.4 ± 0.6 years) (Table 2). Spearman’s rank correlation test did not identify any correlations among SIL-6 and JIL-6 levels at 24 and 72 h, and KSSK and KSSF scores at 4 weeks and at the most recent evaluation (Table 3). No intraoperative complications were observed. However, one patient in the ALBC group developed a superficial wound infection that responded to an oral antibiotic and another ALBC patient had postoperative joint stiffness that required manipulation under general anesthesia. After the described interventions, both patients had an uneventful follow-up period.

**Table 2:** Knee Society Score during the follow-up period

	BC group		ALBC group		*P*	
	4 weeks	Recent evaluation	4 weeks	Recent evaluation	4 weeks	Recent evaluation
		(1.5 ± 0.7 years)		(1.4 ± 0.6 years)		
KSSK	67 (17) 44–76	94 (7) 76–100	61 (12) 45–75	95 (10) 72–100	0.43	0.84
KSSF	5 (15) 0–45	80 (10) 30–100	20 (15) 0–55	80 (15) 55–90	0.08	0.57

Data are presented as mean ± standard deviation for year and median (IQR) and range for KSSK and KSSF

*ALBC* antibiotic-loaded bone cement, *BC* bone cement, *KSSF* Knee Society Score-function score, *KSSK* Knee Society Score-knee score

**Table 3:** Correlations among serum and joint fluid interleukin-6 levels and the Knee Society Score at each time point

Postoperation		SIL-6				JIL-6	
		24 h		72 h		24 h	
		ρ	*p*	ρ	*p*	ρ	*p*
4 weeks	KSSK	0.19	0.24	0.13	0.44	<0.001	0.99
	KSSF	0.07	0.69	0.002	0.99	−0.34	0.05
Recent evaluation	KSSK	−0.02	0.89	−0.09	0.61	0.12	0.49
	KSSF	0.10	0.55	0.18	0.29	0.15	0.39

*JIL-6* joint fluid interleukin-6, *KSSF* Knee Society Score-function score, *KSSK* Knee Society Score-knee score, *SIL-6* serum interleukin-6, *ρ* Spearman’s rank correlation coefficient

## Discussion

Cemented total knee arthroplasty is one of the gold standards of treatment for severe knee OA that provides excellent long-term results. Antibiotics like gentamicin are premixed into bone cement powder by the manufacturer to provide a high local concentration of antibiotics to provide infection prophylaxis after TKA [11, 13]. Based on our review of the literature, this is the first comparative study to demonstrate the potential favorable non-antibacterial effects of antibiotic-impregnated bone cement. Specifically, the results of this non-randomized prospective trial revealed and illuminated the immunomodulatory effect of antibiotic-impregnated bone cement via levels of inflammatory cytokines IL-6 and acute-phase protein responses.

Cytokine activation following major surgery is a well-established phenomenon [1, 2]. IL-6 could be induced by several intraoperative stimuli and its levels inter-related with increased severity of tissue trauma, degree of inflammatory action, and complications [1–3, 17]. Inflammatory reaction may affect postoperative functional outcome in TKA. IL-6 secretion by fibroblasts found in a normal reparative process was associated with an exaggerated proliferation of fibroblast that led to arthrofibrosis [7]. Ugras et al. reported a correlation between intra-articular IL-6 level and functional outcome scores. They concluded that control of local inflammatory response is important for an early functional recovery [6]. Several modalities for reducing postoperative inflammation have been explored. A minimally invasive TKA procedure resulted in less traumatized tissue and a faster recovery of range of motion after TKA [4]. Reducing inflammatory responses via multimodal approaches intraoperatively linked to alterations in catabolic responses delivered a better postoperative outcome [5].

Gentamicin, a broad-spectrum aminoglycoside antibiotic that is available in powder form, is commonly used as a bone cement admixture [9]. The common dosage of 1 g of antibiotic per 40 g of bone cement powder is considered a low dose ratio. An in vivo study demonstrated that gentamicin elusion from bone cement peaked at 24 h after surgery. The level declined thereafter, but continued at a therapeutic level for up to 14 days [18]. Systemic absorption of gentamicin elute from bone cement can be detected during the first 24 h after bone cement implantation [9]. Systemic gentamicin toxicity is also a concern. However, as reports of gentamicin toxicity used high doses of gentamicin for treatment of established periprosthetic joint infection, low-dose gentamicin premixed into bone cement should not cause harmful effects [10, 13]. A study of low-dose tobramycin, an aminoglycoside antibiotic, found that the systemic level was low compared to its peak joint fluid level after total hip arthroplasty [19]. The mechanism by which antibiotics may be able to regulate immunologic responses is not fully understood [20, 21]. Antibiotics could have an effect on normal host cells or alter cellular cytokine production in both normal and septic environments [14]. The mechanism could be an up-regulation or down-regulation of cytokine production at the intracellular level [20]. Gentamicin demonstrated a negative effect on immunomodulating profiles, including cytokine production [15, 21]. In an in vitro septic model, gentamicin was shown to reduce SIL-6 by down-regulation of mRNA transcription via stimulation of toll-like receptors 4 and 6 [22].

The ascending and descending patterns of SIL-6 and JIL-6 levels observed in this study were similar to patterns from previous reports [3, 17, 23, 24]. However, SIL-6 and JIL-6 levels in the ALBC group showed lower levels compared to those of the BC group at the same time points. The potential explanation for our findings is that the local elution of gentamicin could modulate IL-6 secretion, thus modifying IL-6 level in systemic circulation. The mechanism could be an alteration of cellular cytokine production at the TKA site, given that the mean JIL-6 level was approximately 7–8 times the mean SIL-6 level. The immunomodulatory effect of gentamicin may not be immediate, perhaps needing time for the effect to be significantly exhibited. This could explain our finding that SIL-6 levels in the ALBC group showed a significant decrease at 72 h compared to the BC group, but not at 24 h after surgery. Another clinical application from this finding is that SIL-6 should return to normal baseline value within 4 weeks when using ALBC. If SIL-6 does not return to normal within 4 weeks, this could be a red flag for a possible immediate complication like postoperative infection [3, 23, 24].

IL-6 is the principal regulator of the major acute-phase proteins by triggering several body immune response pathways [24–26]. We speculated that a lower level of IL-6 could lead to a lower of level of ESR and CRP, since IL-6 secretion by local stimuli would lead to a multisystem acute-phase protein response [26]. Smith et al. demonstrated that CRP level was one of the predictive factors for postoperative TKA function [8]. The changing pattern of ESR and CRP in our study was in agreement with another recent study [24]. Median (IQR) CRP level in the ALBC group tended to be lower than that of the BC group. The ESR level in the ALBC group followed the same pattern as the CRP level, except at 72 h after surgery, a time point when the ESR level of the ALBC group was higher than that of the BC group. This finding could be due to the fact that ESR and CRP levels are slower than the IL-6 level to react to stimuli, which is consistent with the findings of a previous study [3]. ESR and CRP levels may also be influenced by factors other than IL-6 function.

Alteration of inflammatory cytokine IL-6 after TKA using gentamicin-loaded bone cement was not associated with functional outcomes in our study. Although there was a trend toward a negative correlation between high JIL-6 levels and KSSF at 4 weeks, no significant difference in KSSK and KSSF scores was observed between the two bone cement groups. This was similar to a recent report that described how a minimally invasive TKA variant that was intended to reduce postoperative inflammation provided only short-term functional benefit, but no improved benefit at long-term follow-up [4].

This study has some mentionable limitations. First, the number of patients in each study arm was relatively small. Post-hoc power analysis determined that the sample size should yield a 42 % power to differentiate mean SIL-6 levels between groups. Accordingly, 51 patients in each arm would be needed to attain a power of 80 % at α = 0.05 and β = 20 %. Second, the effect of a postoperative reduction of SIL-6 level on prosthesis survival and functional outcome requires further investigation. Finally and due to ethical concerns, JIL-6 levels were not obtained after 24 h following surgery. As such, the local immunomodulatory effect of gentamicin beyond the 24-h time point is not known.

In conclusion, the results of this study offer compelling evidence that ALBC significantly decreases SIL-6 levels at 72 h following TKA, when compared to SIL-6 levels in TKA patients who received conventional bone cement. Antibiotic-impregnated bone cement may produce favorable immunomodulatory effects by altering inflammatory cytokine production in addition to its co-primary role as a grouting agent and a prophylaxis against infection. The functional benefits of ALBC require further investigation.
